# Relationship of hyperlipidemia to comorbidities and lung function in COPD: Results of the COSYCONET cohort

**DOI:** 10.1371/journal.pone.0177501

**Published:** 2017-05-15

**Authors:** Kathrin Kahnert, Tanja Lucke, Rudolf M. Huber, Jürgen Behr, Frank Biertz, Anja Vogt, Henrik Watz, Peter Alter, Sebastian Fähndrich, Robert Bals, Rolf Holle, Stefan Karrasch, Sandra Söhler, Margarethe Wacker, Joachim H. Ficker, Klaus G. Parhofer, Claus Vogelmeier, Rudolf A. Jörres, on behalf of the COSYCONET consortium

**Affiliations:** 1 Department of Internal Medicine V, University of Munich, Comprehensive Pneumology Center, Member of the German Center for Lung Research, Munich, Germany; 2 Institute and Outpatient Clinic for Occupational, Social and Environmental Medicine, Ludwig-Maximilians-Universität München, Munich, Germany; 3 Institute for Biostatistics, Hannover Medical School, Hannover, Germany; 4 Stoffwechselambulanz, Klinik und Poliklinik der Universität München, Munich, Germany; 5 Pulmonary Research Institute at LungenClinic Grosshansdorf, Airway Research Center North, Member of the German Center for Lung Research, Grosshansdorf, Germany; 6 Department of Medicine, Pulmonary and Critical Care Medicine, University Medical Center Giessen and Marburg, Philipps-University Marburg, Germany, Member of the German Center for Lung Research (DZL), Marburg, Germany; 7 Department of Internal Medicine V – Pulmonology, Allergology, Respiratory Intensive Care Medicine, Saarland University Hospital, Homburg, Germany; 8 Institute of Health Economics and Health Care Management, Helmholtz Zentrum München (GmbH) - German Research Center for Environmental Health, Member of the German Center for Lung Research, Comprehensive Pneumology Center Munich (CPC-M), Neuherberg, Germany; 9 Institute of Epidemiology I, Helmholtz Zentrum München - German Research Center for Environmental Health, Neuherberg, Germany; 10 ASCONET Study Coordination Office, University of Marburg, Marburg, Germany; 11 Department of Respiratory Medicine, Allergology and Sleep Medicine, General Hospital Nuernberg, Paracelsus Medical University, Nuernberg, Germany; 12 Department of Internal Medicine IV, University of Munich, Munich, Germany; National and Kapodistrian University of Athens, GREECE

## Abstract

Although hyperlipidemia is common in COPD, its relationship to comorbidities, risk factors and lung function in COPD has not been studied in detail. Using the baseline data of the COSYCONET cohort we addressed this question. Data from 1746 COPD patients (GOLD stage 1–4; mean age 64.6 y, mean FEV1%pred 57%) were evaluated, focusing on the comorbidities hyperlipidemia, diabetes and cardiovascular complex (CVC; including arterial hypertension, cardiac failure, ischemic heart disease). Risk factors comprised age, gender, BMI, and packyears of smoking. The results of linear and logistic regression analyses were implemented into a path analysis model describing the multiple relationships between parameters. Hyperlipidemia (prevalence 42.9%) was associated with lower intrathoracic gas volume (ITGV) and higher forced expiratory volume in 1 second (FEV_1_) when adjusting for its multiple relationships to risk factors and other comorbidities. These findings were robust in various statistical analyses. The associations between comorbidities and risk factors were in accordance with previous findings, thereby underlining the validity of our data. In conclusion, hyperlipidemia was associated with less hyperinflation and airway obstruction in patients with COPD. This surprising result might be due to different COPD phenotypes in these patients or related to effects of medication.

## Introduction

Hyperlipidemia is a major risk factor for cardiovascular diseases which are common comorbidities in patients with chronic obstructive pulmonary disease (COPD) [1], but there are only limited studies on its role in COPD itself [2, 3]. Although most comorbidities are associated with increased mortality, retrospective analyses revealed a decreased incidence of pneumonia and reduced mortality in COPD patients with hyperlipidemia [4, 5]. Thus the role of hyperlipidemia remains unclear and its relationship to other comorbidities, risk factors and pulmonary function has to be studied in more detail.

In subjects with metabolic syndrome and hyperlipidemia but no obvious lung disease a restrictive spirometric lung function pattern was observed [6–8], but this has not been verified e.g. by bodyplethysmography. The relation of hyperlipidemia to lung function in COPD is unclear but of interest owing to physiological findings. Lipoproteins can influence blood rheology, including plasma viscosity, aggregation and deformation of erythrocytes [9, 10], and *in vitro* they led to accumulation of erythrocytes in the pulmonary capillary bed [11]. Thus it has been hypothesized that a higher capillary red cell volume would lead to an increase in the diffusing capacity for carbon monoxide (TLCO). At least in lung-healthy subjects, however, there was no difference in TLCO between subjects with and without hyperlipidemia [11–13].Whether this also applies to patients with COPD is not known.

Based on these considerations the aim of our study was to analyze the relationship of hyperlipidemia to lung function, risk factors and comorbidities linked to hyperlipidemia in patients with COPD. For this purpose we used data from the German COSYCONET (“COPD and SYstemic consequences-COmorbidities NETwork”) COPD cohort [14].

## Material and methods

### Study population

The analysis was based on the baseline data set (visit 1) of COSYCONET which is a multi-center study focusing on the role of comorbidities in COPD [14]. Only patients of COPD severity GOLD 1–4 [15] with complete data on forced expiratory volume in 1 second (FEV_1_), intrathoracic gas volume (ITGV) and transfer coefficient for carbon monoxide (KCO), as well as on age, gender, packyears of smoking, body-mass index (BMI) and the comorbidities hyperlipidemia, diabetes, and cardiovascular complex (see below) were included. This resulted in a study population of n = 1746 out of 2741 patients recruited into COSYCONET [14]. The study had been approved by the ethical committees of all study centers and all patients gave their written informed consent.

### Ethics approval and consent to participate

All assessments were approved by the central (Marburg (Ethikkommission FB Medizin Marburg) and local (Bad Reichenhall (Ethikkommission bayerische Landesärztekammer); Berlin (Ethikkommission Ärztekammer Berlin); Bochum (Ethikkommission Medizinische Fakultät der RUB); Borstel (Ethikkommission Universität Lübeck); Coswig (Ethikkommission TU Dresden); Donaustauf (Ethikkommission Universitätsklinikum Regensburg); Essen (Ethikkommission Medizinische Fakultät Duisburg-Essen); Gießen (Ethikkommission Fachbereich Medizin); Greifswald (Ethikkommission Universitätsmedizin Greifswald); Großhansdorf (Ethikkommission Ärztekammer Schleswig-Holstein); Hamburg (Ethikkommission Ärztekammer Hamburg); MHH Hannover / Coppenbrügge (MHH Ethikkommission); Heidelberg Thorax/Uniklinik (Ethikkommission Universität Heidelberg); Homburg (Ethikkommission Saarbrücken); Immenhausen (Ethikkommission Landesärztekammer Hessen); Kiel (Ethikkommission Christian-Albrechts-Universität zu Kiel); Leipzig (Ethikkommission Universität Leipzig); Löwenstein (Ethikkommission Landesärztekammer Baden-Württemberg); Mainz (Ethikkommission Landesärztekammer Rheinland-Pfalz); München LMU/Gauting (Ethikkommission Klinikum Universität München); Nürnberg (Ethikkommission Friedrich-Alexander-Universität Erlangen Nürnberg); Rostock (Ethikkommission Universität Rostock); Berchtesgadener Land (Ethikkommission Land Salzburg); Schmallenberg (Ethikkommission Ärztekammer Westfalen-Lippe); Solingen (Ethikkommission Universität Witten-Herdecke); Ulm (Ethikkommission Universität Ulm); Würzburg(Ethikkommission Universität Würzburg)) ethical committees and written informed consent was obtained from all patients. The study was conducted from September 2011 to December 2013 within the COSYCONET framework (ClinicalTrials.gov, Identifier: NCT01245933) [14]. The approval by the central ethics committee (University of Marburg) and the ethics committees of all other studies centers (including University of Munich) comprises the statement that the study can be conducted. This includes not only the collection of data but also the permission that the analyses of the data outlined in the study protocol, as well as study questions developed on the basis of accumulated experience and the collected data, can be performed. An additional approval is only required if additional data are collected which were not part of the initial approval or if patient-sensitive analyses are to be performed, such as genetic analyses of collected samples without previous approval by the patient. In their written informed consent the patients also agreed to the scientific evaluation of the collected data. The specific aim of the present study, which comprised the analysis of three comorbidities and selected lung function parameters, is therefore implicitly included in the approval by the ethics committees. Naturally, in such a large cohort study including multiple parameters not all specific study questions can be explicitly formulated from the beginning. The question of the present study was contained in the bullet point “zu untersuchen, ob die Kombination funktioneller Indizes, systemischer Marker und klinischer Diagnosen es erlaubt, bislang unbekannte Phänotypen der COPD zu definieren, die möglicherweise einer unterschiedlichen Verlaufskontrolle und Therapie bedürfen”(translated: to study whether the combination of functional indices, systemic markers and clinical diagnoses allows the definition of novel phenotypes of COPD which possibly require a different monitoring over time and therapy).

### Methods

Protocol and methods have been described previously [14]. To facilitate the comparison with previous cohorts we described the characteristics of our study population by a panel of parameters as well as the conventional categorizations (1–4 and ABCD) according to GOLD [15]. Moreover we used the conventional percentages of mean predicted values based on the fact that the data on the deviations allowing for a lower limit of predicted was heterogeneous among the predictions equations for the different parameters used. For the present analysis comorbidities were assumed if either patients reported a doctor-based diagnosis, irrespective of medication, or in the absence of a report, if disease-specific medication was identified; the details of the procedure and the medication are given in a previous publication [16]. These definitions were named „extended definitions“. Since in cardiovascular diseases medication often is not specific for a single diagnosis but for two or three, we combined the three comorbidities “arterial hypertension”, “cardiac failure” and “ischemic heart disease” into „cardiovascular complex” [16]. The analysis was restricted to three comorbidities known to be closely linked to each other based on clinical observations. Moreover in these comorbidities disease-specific medication could be used to higher degree than in a variety of other comorbidities; the complete list of comorbidities is given in our previous publication [14].

Lung function comprised spirometry, body plethysmography and carbon monoxide (CO) diffusing capacity [14]. In addition to FEV_1_, ITGV and KCO, for the description of the population the forced vital capacity (FVC), the ratio FEV_1_/FVC and transfer factor for CO (TLCO) were chosen, each as percent of predicted. For the path analysis FEV_1_, ITGV and KCO were taken as representatives of the domains obstruction, hyperinflation and gas exchange limitation. Predicted values of FEV_1_, FEV_1_/FVC, FVC were taken from GLI [17], of ITGV from EGKS [18], and of TLCO and KCO from van der Lee et al. [19]. As major risk factors we included BMI, age, gender and packyears.

### Statistical analysis

Data are presented as mean values and standard deviations (SD). Comparisons between the two groups with and without hyperlipidemia were performed with the unpaired t-test irrespective of potential small deviations from normality (Kolmogorov-Smirnov-test) which commonly have no major impact on the test result. However, to be on the safe side and to check whether these deviations affected the result we additionally employed the Mann-Whitney-U-test and explicitly give the results for both types of tests. Categorical variables were compared between groups using cross-tabulation and the chi-square-test statistics.

Next the association between variables was evaluated by standard multivariate linear and binary logistic regression analyses comprising one dependent and multiple independent variables. These types of analyses are however limited to describe complex relationships in networks that can be represented by only two types of variables: dependent and independent. A potential relationship between dependent variables as well as the possibility that the same variable is both dependent and independent can be modeled by path analysis [20]. All analyses were performed using SPSS Statistics 23 (IBM Corp., Armonk, NY, USA) and AMOS (IBM Corp., Armonk, NY, USA). Statistical significance was assumed for p<0.05.

## Results

### Patients’ characteristics

**Fig 1:**
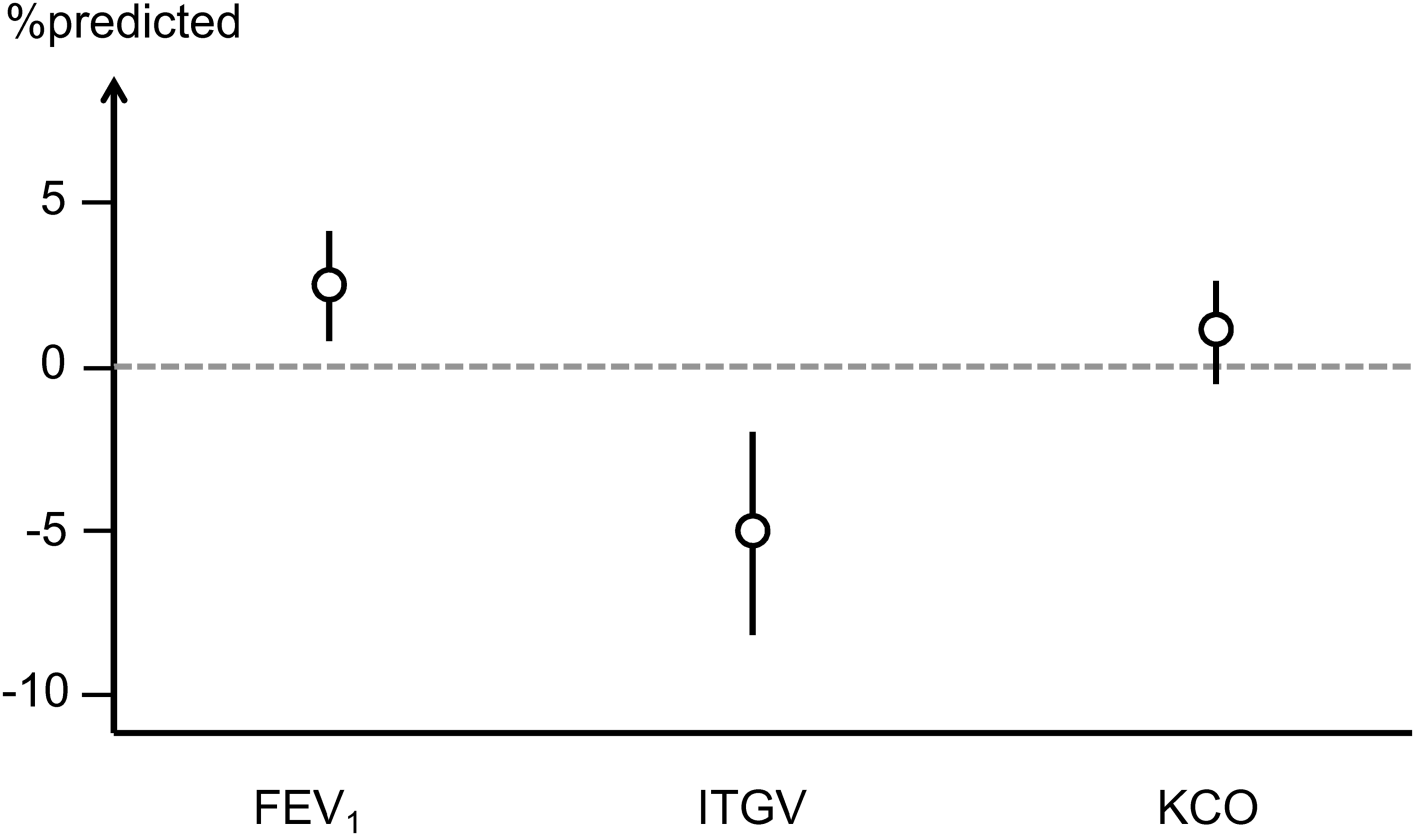
Adjusted effects of hyperlipidemia on lung function. The figure shows the differences between patients with and without hyperlipidemia for three selected lung function parameters representing airway obstruction, lung volume and alveolar gas exchange. These differences are based on multivariate regression analyses adjusting for age, gender, BMI and packyears, as major confounders some of which were different between groups. The circles represent mean values and the vertical bars 95% confidence intervals, showing that even after adjustment there were significant (p<0.05) differences in FEV_1_ and ITGV.

Table 1 shows the patients’ characteristics (n = 1746) stratified for hyperlipidemia. In all parameters except for FVC%pred, FEV_1_/FVC and TLC%pred there were significant differences between the two groups, in both parametric and non-parametric testing. For comparison the values for the total COSYCONET study population (GOLD 1–4, n = 2238) are given in S1 Table; patients with and without hyperlipidemia again significantly differed in all parameters except FEV_1_/FVC and TLC%pred. For selected lung function parameters the differences between hyperlipidemia groups remained significant after adjustment for risk factors and are illustrated in Fig 1.

**Table 1:** Baseline characteristics of the subgroups with and without hyperlipidemia.

Parameter	All patients	Hyperlipidemia **(extended def.)**	Non-Hyperlipidemia	p-values
**N (%)**	1746	749 (42.9%)	997 (57.1%)	-
**Gender (m/f)**	1092/654	498/251	594/403	p = 0.003*
**Age (y)**	64.6 (±8.4)	65.8 (±7.8)	63.8 (±8.8)	p<0.001*
**BMI (kg/m^2^)**	26.8 (±5.3)	27.6 (±5.2)	26.2 (±5.2)	p<0.001*
**Waist circ. (cm)**	99.6 (±15.6)	102.1 (±15.3)	97.7 (±15.5)	p<0.001*
**Packyears**	49.2 (±35.8)	52.4 (±37.8)	46.7 (±34.0)	p = 0.001*
**Hb (mg/dl)**	14.71 (±1.34)	14.59 (±1.42)	14.79 (±1.29)	p = 0.003*
**Creatinine (mg/dl)**	0.90 (±0.24)	0.93 (±0.26)	0.87 (±0.22)	p<0.001*
**Triglycerides (md/dl)**	141.5 (±106.6)	156.9 (±111.1)	129.9 (±101.7)	p<0.001*
**Total cholesterol (mg/dl)**	214.2 (±43.4)	209.6 (±48.2)	217.8 (±39.1)	p<0.001*
**LDL (mg/dl)**	126.8 (±38.0)	123.1 (±41.5)	129.5 (±34.9)	p≤0.001*
**HDL (mg/dl)**	64.1 (±20.9)	61.9 (±20.8)	65.8 (±20.9)	p<0.001*
**FEV_1_%pred**	56.9 (±19.1)	58.6 (±18.8)	55.6 (±19.3)	p = 0.001*
**FEV_1_/FVC**	54.7 (±13.8)	55.0 (±13.6)	54.6 (±19.3)	p = 0.451
**FVC%pred**	78.3 (±19.1)	77.6 (±19.3)	78.9 (±19.0)	p = 0.166
**TLC%pred**	110.9 (±29.8)	109.8 (±29.0)	111.7 (±30.3)	p = 0.188
**RV%pred**	153.9 (±45.3)	148.0 (±42.5)	158.4 (±46.9)	p<0.001*
**ITGV%pred**	149.4 (±35.0)	144.1 (±33.9)	153.3 (±35.2)	p<0.001*
**VA (liter)**	4.7 (±1.6)	4.8 (±1.6)	4.7 (±1.7)	p = 0.316
**TLCO%pred**	50.6 (±19.7)	52.1 (±19.1)	49.5 (± 20.1)	p = 0.006*
**KCO%pred**	64.0 (±22.4)	66.5 (±22.1)	62.1 (±22.6)	p<0.001*
**GOLD 1/2/3/4**	232/934/24/584	106/367/242/34	126/438/363/70	p = 0.022*
**GOLD A/B/C/D**	199/934/24/582	82/396/6/264	117/538/18/318	p = 0.123

The table shows mean values and standard deviations or absolute numbers. Lung function parameters are given in terms of %predicted, except for alveolar volume, VA, which is given in liters. Column 4 shows the results of comparisons between the hyperlipidemia group (extended definition) and the complementary group of non-hyperlipidemia patients. The comparisons between groups were performed by unpaired t-tests, either for equal or unequal variances depending on the data, or by chi-square-tests in the case of categorical variables. The results of t-tests were checked by the Mann-Whitney-U-test to accommodate for deviations from normality; the results of both approaches were qualitatively equivalent. Significant (p<0.05) differences are marked with (*).

**Fig 2:**
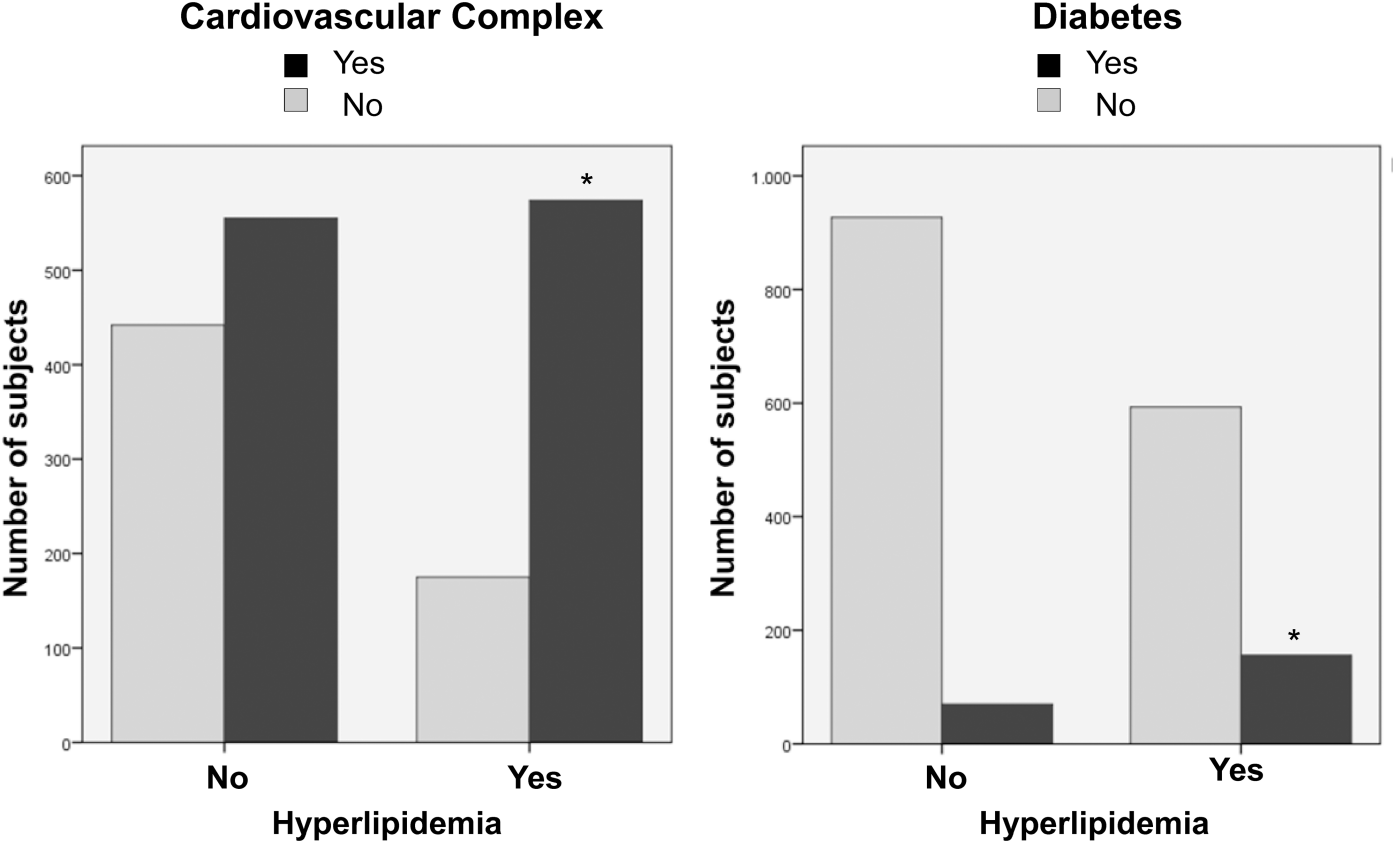
Prevalence of hyperlipidemia versus diabetes and cardiovascular complex. Diabetes and cardiovascular complex were associated with hyperlipidemia. Significant differences (p<0.001) were marked with (*).

Fig 2 shows the prevalence of hyperlipidemia in patients with and without diabetes or cardiovascular complex. Both were significantly (p<0.001 each) associated with hyperlipidemia. Overall these results showed that (a) our observations were in accordance with other cohorts and that (b) it would be reasonable to implement the relationships between comorbidities, and not only their relation to risk factors or lung function, into a statistical model.

### Results of path analysis models

We first performed standard multivariate linear and logistic regression analyses (see Tables A-C in S1 Text). Based on their results we constructed a sequence of path analysis models which allowed the description of more complex relationships, particularly between dependent variables. To build a final model in a systematic way, two preliminary models were evaluated (see S1 and S2 Figs). In this model the variables were arranged into three „layers“: on top the risk factors (BMI, age, gender, packyears), as intermediates the comorbidities (diabetes, hyperlipidemia, cardiovascular complex), and at the bottom lung function (ITGV%pred, FEV_1_%pred, KCO%pred).

#### Final model comprising risk factors, comorbidities and lung function

**Fig 3:**
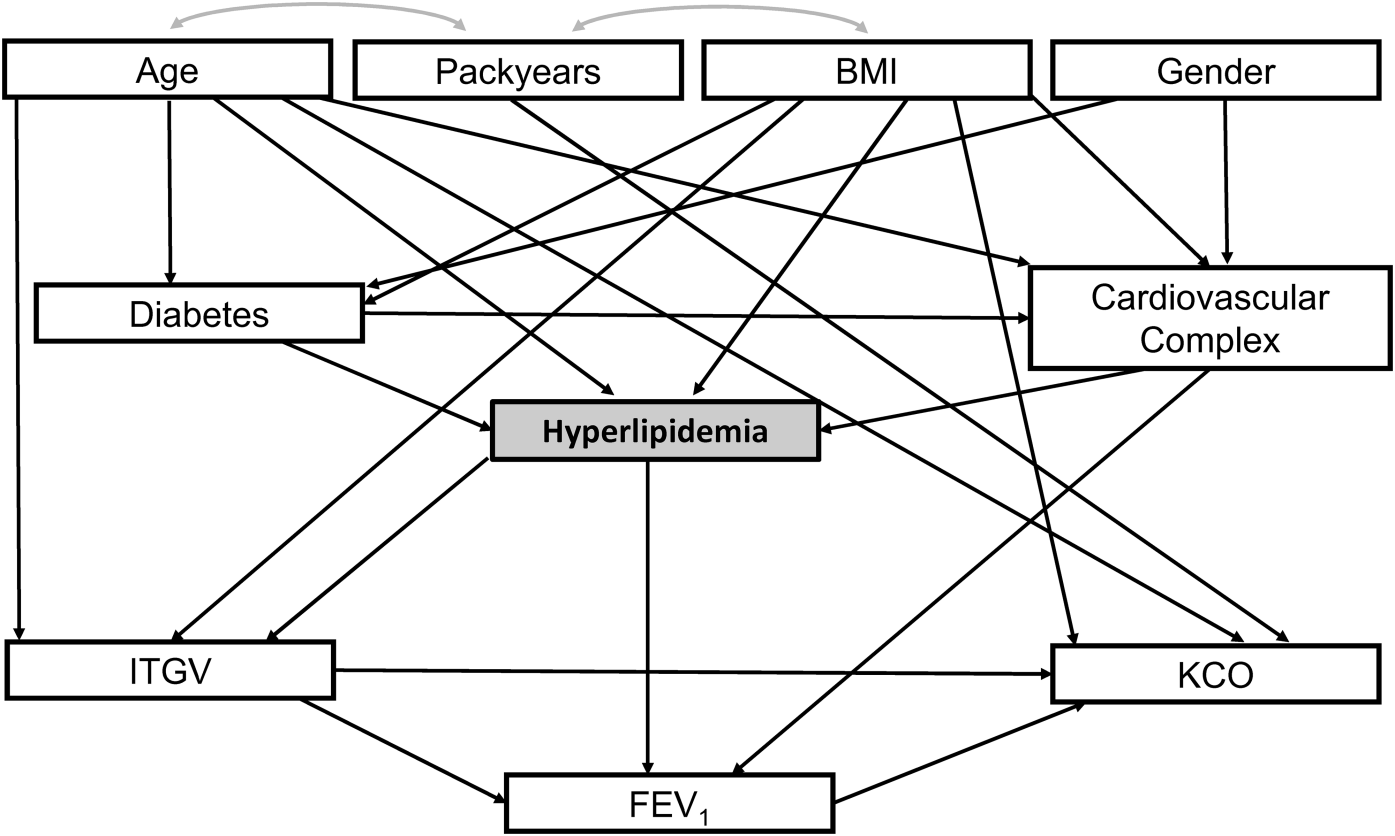
Results of path analysis. Final path analysis model comprising three layers: risk factors, comorbidities and lung function parameters. The structure only contains relationships which turned out to be statistically significant (p<0.05 each). Error terms of dependent variables have been omitted for the sake of clarity. Correlations between the independent variables are indicated by arched arrows.

The final model (Fig 3) was constructed as a composite of the two preliminary models (S1 and S2 Figs). Based on the regression results (Table C in S1 Text) we additionally introduced a number of relationships between comorbidities and lung function but kept only those which turned out to be statistically significant, i.e. that between hyperlipidemia and ITGV and that between cardiovascular complex and FEV_1_. Parameter values and significance levels of the final model are shown in Table 2. The model fitted the data with a chi-squared of 18.79 at 21 degrees of freedom (p = 0.60; see above) and was also well-fitting according to the bootstrap procedure using 2000 samples and the Bollen-Stine method (chi-squared 21.54, p = 0.61).

**Table 2:** Results of the final path analysis model.

Regression			Estimate	S.E.	C.R.	Standardized Estimate	p-value
Diabetes	←	BMI	.059	.007	8.311	.196	p<0.001
Diabetes	←	Gender	-.102	.014	-7.069	-.147	p<0.001
Diabetes	←	Age	.016	.007	2.361	.053	p = 0.018
Cardiovascular complex	←	BMI	.072	.010	7.350	.168	p<0.001
Cardiovascular complex	←	Diabetes	.186	.026	7.186	.130	p<0.001
Cardiovascular complex	←	Gender	-.100	.023	-4.335	-.101	p<0.001
Cardiovascular complex	←	Age	.081	.010	8.052	.184	p<0.001
Dyslipidemia	←	Diabetes	.224	.034	6.574	.152	p<0.001
Dyslipidemia	←	Cardiovascular complex	.169	.024	6.899	.163	p<0.001
Dyslipidemia	←	BMI	.036	.011	3.375	.080	p<0.001
Dyslipidemia	←	Age	.023	.011	2.191	.051	p = 0.028
ITGV	←	BMI	-.314	.022	-14.415	-.313	p<0.001
ITGV	←	Age	-.105	.022	-4.663	-.102	p<0.001
ITGV	←	Dyslipidemia	-.121	.051	-2.363	-.054	p = 0.018
FEV_1_	←	ITGV	-.521	.019	-28.093	-.533	p<0.001
FEV_1_	←	Cardiovascular complex	-.242	.046	-5.211	-.106	p<0.001
FEV_1_	←	Dyslipidemia	.121	.046	2.635	.055	p = 0.008
KCO	←	ITGV	-.193	.027	-7.200	-.193	p<0.001
KCO	←	FEV_1_	.234	.025	9.206	.228	p<0.001
KCO	←	Packyears	-.110	.021	-5.304	-.109	p<0.001
KCO	←	BMI	.233	.021	10.839	.232	p<0.001
KCO	←	Age	.074	.021	3.523	.073	p<0.001
Covariances							
BMI	↔	Packyears	.143	.029	4.969	.116	p<0.001
Packyears	↔	Age	.099	.029	3.465	.082	p<0.001

The upper panel refers to the directed arrows (linear regression terms) depicted in Fig 2. The left part of this panel lists the arrows shown in this figure, the right part shows the results of the corresponding statistical tests. The first column of the right part shows the non-standardized estimate of the respective regression coefficient, the second column the standard error of this coefficient (S.E.), the third column the ratio of these two (critical ratio. C.R.) which is used for significance testing. The forth column shows the standardized estimates of the regression coeffients shown in the first column. The last column shows the significance level based on the asymptotically distribution-free estimation procedure of AMOS. All coefficients were also significant when using the standard maximum likelihood estimation procedure despite the deviations from normal distribution for nearly all variables. The standardized estimates are given since they allow for the evaluation of direct and indirect effects: direct effects from one variable onto the other are given by the respective standardized regression coefficient, whereas indirect effects mediated through a third variable are given by the multiplication of the two standardized regression coefficients between the respective variables. The lower panel shows the covariances (bidirectional arrows) between the risk factors that were part of the model, as well as the respective standard errors, critical ratios and significance levels. The standardized covariances represent the respective correlation coefficients.

## Discussion

In the present analysis we investigated the relationship between risk factors, comorbidities and lung function in a large cohort of patients with COPD. The comorbidities comprised a combination of cardiovascular diseases and diabetes, as well as hyperlipidemia [2, 3]. Its prevalence was 42.9% according to self-reported doctors’ diagnoses and disease-specific medication. Based on linear and logistic regression results an integrative path analysis model was built that illustrated the place of hyperlipidemia in the network of risk factors, other comorbidities and lung function. Hyperlipidemia was dependent on age, BMI, diabetes and cardiovascular diseases. Even after adjustment for confounders it was associated with lower ITGV and higher FEV_1_, beyond the direct and indirect links from BMI and age. This apparently protective effect might be related to the phenotype of COPD as well as beneficial effects of medication targeted at hyperlipidemia. In our analyses we required complete data sets and therefore had to exclude a number of patients. However comparison of the descriptive Table 1 and S1 Table does not indicate a selection bias introduced by this.

To understand the relationship between the different entities we first performed multivariate regression analyses comprising the risk factors age, packyears, BMI and gender, the three comorbidities and a variety of lung function parameters. For path analysis we focused on single representatives of obstruction (FEV_1_%pred), hyperinflation (ITGV%pred) and gas exchange (KCO%pred). Among the comorbidities a combined entity “cardiovascular complex” was defined comprising arterial hypertension, cardiac failure and ischemic heart disease. The comorbidities depended on age, BMI and partially on gender (Table A in S1 Text), while lung function depended on BMI, age and gender, diffusing capacity additionally on packyears (Table B in S1 Text). Diabetes was related to lung volume and diffusing capacity, cardiovascular complex to obstruction and lung volume, and hyperlipidemia to all three lung function parameters (Table C in S1 Text).

Thus hyperlipidemia was linked to risk factors as well as lung function. It was associated with relatively better FEV_1_, while diabetes and cardiovascular complex were linked to relatively lower FEV_1_. The observation regarding hyperlipidemia and FEV_1_ appeared to be in contrast to findings in obese lung-healthy subjects [21] but in COPD the situation might be more complicated due to the presence of risk factors influencing both lung function and comorbidities. To analyse the complex network of direct and indirect effects we used the statistical procedure of path analysis.

Path analysis is a well-founded statistical procedure widely used in empirical social sciences and econometrics [20]; it has also been used in medical studies to evaluate complex associations [22, 23]. Essentially it is an extension of multivariate regression allowing for hierarchical relationships as well as bypassing these hierarchies and thus the quantification of both direct and indirect effects all of which can be visualized graphically; indirect effects are those mediated through other variables (see S1 Text). The possibility to introduce relationships between dependent variables accounts for known or suspected links between them, in addition to the “downward” relationships quantified by conventional regression. We first analysed two preliminary models which comprised risk factors and either comorbidities (S1 Fig) or lung function (S2 Fig). These models showed that (a) the assumed additional relationships were statistically valid and (b) that no further significant relationships could be demonstrated. Irrespective of this it has to be kept in mind that path analysis is a hypothesis-driven procedure and that in general there may be statistically equivalent models. The choice between them has to be based on physiological and clinical knowledge. On the other hand the procedure is capable of excluding models that do not adequately describe the data [20].

The final model (Fig 3) was constructed as overlay of the preliminary models. Additionally we introduced relationships between comorbidities and lung function taking into account the results of the respective regression analysis (Table C in S1 Text). Only the relationships between hyperlipidemia, FEV_1_%pred and ITGV%pred remained as significant and were kept in the model. It seems remarkable that these direct links from hyperlipidemia were still relevant when multiple confounders were taken into account but this was in accordance with comparisons adjusting for risk factors (Fig 1). The link from hyperlipidemia to ITGV acted in parallel to the direct effect of BMI and had the same sign, i.e. hyperlipidemia was associated with less hyperinflation. This reduction of ITGV might reflect an additional mechanical influence in obese subjects that is not adequately described by BMI, such as a different distribution of body mass. To check this possibility we repeated the analysis with waist circumference as predictor instead of BMI. The overall model fit was still acceptable, and the links between hyperlipidemia and ITGV%pred as well as FEV_1_%pred remained significant. Therefore we considered BMI as adequate in the model.

Among the comorbidities of COPD known to be linked to hyperlipidemia, diabetes and cardiovascular diseases are the most prevalent ones [3]. We therefore restricted the analysis to these two conditions. One of the advantages was that these comorbidities could also be evaluated via analysis of disease-specific medication in addition to patients’ report [16]. This however required that the three diseases arterial hypertension, cardiac failure and ischemic heart disease were combined into to a single entity termed “cardiovascular complex”, since many medications are specific for a combination of these diseases but not for a single one. Comparing to reported diagnoses, the prevalence for diabetes increased through the consideration of specific medication from 12.6 to 12.9%, for the cardiovascular complex from 60.0 to 64.7%, and for hyperlipidemia from 37.9 to 42.9%. Probably the estimates including the information from medication are more reliable than those based on reports only. When using the latter, the overall fit of the model was reduced but still significant; importantly, the links between hyperlipidemia, ITGV and FEV_1_ remained significant. This indicates that the extended definitions of comorbidities (a) improved the results and (b) did not introduce artefacts compared to the reported diagnoses.

As risk factors we considered age and gender, as well as packyears and BMI, which were either independent of the individual behaviour, or linked to lifestyle. All of them are known to be correlated with comorbidities and lung function, and indeed their association with cardiovascular diseases was consistent with previous findings [24]. The same was true for the associations between BMI and hyperlipidemia [25] or diabetes [26], or between diabetes and cardiovascular diseases [27], or between diabetes, cardiovascular diseases and hyperlipidemia [3]. This suggests that the COSYCONET data set did not deviate from other data sets in a significant way, irrespective of the diagnosis of COPD. In particular this refers to the link between hyperlipidemia and lung volume which turned to be robust in various statistical analyses. The comorbidities were part of the final model in order to include as much as possible of the known confounders and thereby to identify both direct and indirect associations of hyperlipidemia via their standardized effects (see legend to Table 2). For example, hyperlipidemia was directly linked to age (Table 2; standardized effect 0.045; see also Fig 2) but also indirectly linked to age via diabetes as well as cardiovascular complex which corresponded to an overall indirect effect of 0.039. Thus direct and indirect effects were of similar magnitude. This type of quantification is possible only with path analysis through multiplication of the respective standardized coefficients.

The findings regarding lung volume fit into the hypothesis that COPD patients with higher BMI and/or hyperlipidemia more often show a COPD phenotype of obstructive bronchitis rather than emphysema. We did not have such phenotype information, e.g. based on CT scans, and the pattern of lung function alterations did not uniquely allow the differentiation. The observed inverse association between ITGV and FEV_1_ (Table 2) is known but not specific for emphysema [28], and the relationship between BMI and relatively higher FEV_1_ is consistent with less emphysema in patients of higher weight [29]. The direct link from packyears to lower KCO possibly reflected the degree of emphysema, thereby abolishing a potential effect of dyslipidemia (Fig 1), and the association of higher ITGV with lower KCO (Table 2) their known volume-based relationship. As TLCO is directly proportional to lung volume we preferred the use of KCO, although KCO also does not fully normalize for volume. Moreover the model fit with KCO was superior to that with TLCO.

The positive association between hyperlipidemia and FEV_1_ (Table 2) might be surprising at the first view but is not necessarily in conflict with findings that lung-healthy subjects with hyperlipidemia/metabolic syndrome showed a reduced FEV_1_, since we studied patients with COPD, i.e. inflammatory lung disease. Remarkably, patients with COPD and hyperlipidemia appear to have a better outcome in pneumonia-related exacerbations and mortality [4, 5]. It may also be noteworthy that several studies described an anti-inflammatory action of simvastatin [30], an improved FEV_1_ in patients undergoing this therapy [31] a protective effect against the development of emphysema [32], in accordance with the, on average, better FEV_1_ and KCO in our patients (Table 1). Whether hyperlipidemia-related inflammation favours a specific phenotype of COPD is not known; for diabetes CT data indicate a predominant non-emphysematous type of COPD [33]. Although the differences of lung function parameters between both groups were small, they might become relevant in case of exacerbations.

The diagnosis of hyperlipidemia used in our analyses was based on patients’ reports and medication, while the effectiveness of their lipid-lowering therapy was illustrated by the comparison of patients with and without the diagnosis of hyperlipidemia (Table 1 and S1 Table). This was the reason why we could not use lipids for the definition of hyperlipidemia and did not include them into the path analysis. Overall 23.8% of patients received hyperlipidemia-specific medication [20], the others either non-specific medication and/or dietetics recommendations. When ITGV%pred was evaluated with BMI, age, gender and packyears as confounders, it turned out to be still significantly reduced for both patients with specific and non-specific medication. These observations show that the major difference in patients with the diagnosis of hyperlipidemia compared to those without was the presence of medication and not an increase in lipid levels. It is therefore tempting to attribute our findings to effects of medication.

One of the limitations of our study was that we could characterize the population by conventional lipid parameters but hardly include these in the analyses since they apparently were affected by therapy. A similar situation occurred for cardiovascular diseases. We therefore preferred not to use biomarkers in this analysis. The identification of comorbidities was based on patients’ reports only but we tried to alleviate this limitation as much as possible by using the extended, medication-based definitions. Moreover we did not have detailed information on the phenotype of COPD, e.g. from CT scans. The strength of the study was the large data set, the possibility to evaluate three comorbidities by analysis of medication, and the use of path analysis beyond conventional multivariate regression. The statistical evaluation of the model depended on assumptions on data distribution which were not met with our data, but we used asymptotically distribution-free estimation in a sufficiently large data set [24] and the fit was confirmed by other estimation procedures. Furthermore we incorporated into the final model as much as possible of the already known associations thereby aiming to describe the role of hyperlipidemia in COPD in the whole context of other alterations.

## Conclusion

Using the large baseline data set of the German COPD cohort COSYCONET we evaluated the relationship between risk factors, hyperlipidemia, diabetes, a combination of cardiovascular diseases and lung function. Hyperlipidemia was associated with lower ITGV and higher FEV_1_ even if its direct and indirect links to diabetes and cardiovascular disease, or age, gender and BMI were taken into account, and there was no hint towards an impairment of lung function associated with hyperlipidemia, similar to that previously found for diabetes. The result was statistically robust within a path analysis model and multivariate regression analyses suggesting that COPD patients with hyperlipidemia showed less lung hyperinflation and airway obstruction than those without hyperlipidemia. Whether this reflected differences in COPD phenotype or was related to other factors including medication remains to be clarified.

## Supporting information

S1 Fig Premliminary path analysis model 1. Preliminary path analysis model comprising two layers, on the top risk factors and as intermediate layer comorbidities. All of the relationships shown were statistically significant (p<0.05 each). Error terms of dependent variables have been omitted for the sake of clarity. There were no significant correlations between the independent variables.
(TIF)

S2 Fig Preliminary path analysis model 2. Preliminary path analysis model comprising two layers, on the top risk factors and as bottom layer lung function parameters. All of the relationships shown were statistically significant (p<0.05 each). Error terms of dependent variables have been omitted for the sake of clarity. There were no significant correlations between the independent variables.
(TIF)

S1 Table Baseline characteristics of the subgroups with and without hyperlipidemia (n = 2238, total cohort GOLD 1–4). The table shows mean values and standard deviations or absolute numbers. Lung function parameters are given in terms of %predicted, except for alveolar volume, VA, which is given in liters. Column 4 shows the results of comparisons between the hyperlipidemia group (extended definition) and the complementary group of non-hyperlipidemia patients. The comparisons between groups were performed by unpaired t-tests, either for equal or unequal variances depending on the data, or by chi-square-tests in the case of categorical variables. The results of t-tests were checked by the Mann-Whitney-U-test to accommodate for deviations from normality; the results of both approaches were qualitatively equivalent. Significant (p<0.05) differences are marked with (*).
(DOCX)

S1 Text Supplement/ Supporting information. (DOCX)

S1 File Funding/Support. (DOCX)
