# Supplementary figures and images for: Relationship of hyperlipidemia to comorbidities and lung function in COPD: Results of the COSYCONET cohort
Source: PLoS One. 2017 May 15;12(5):e0177501. doi: 10.1371/journal.pone.0177501 (PMC5432186; doi:10.1371/journal.pone.0177501)

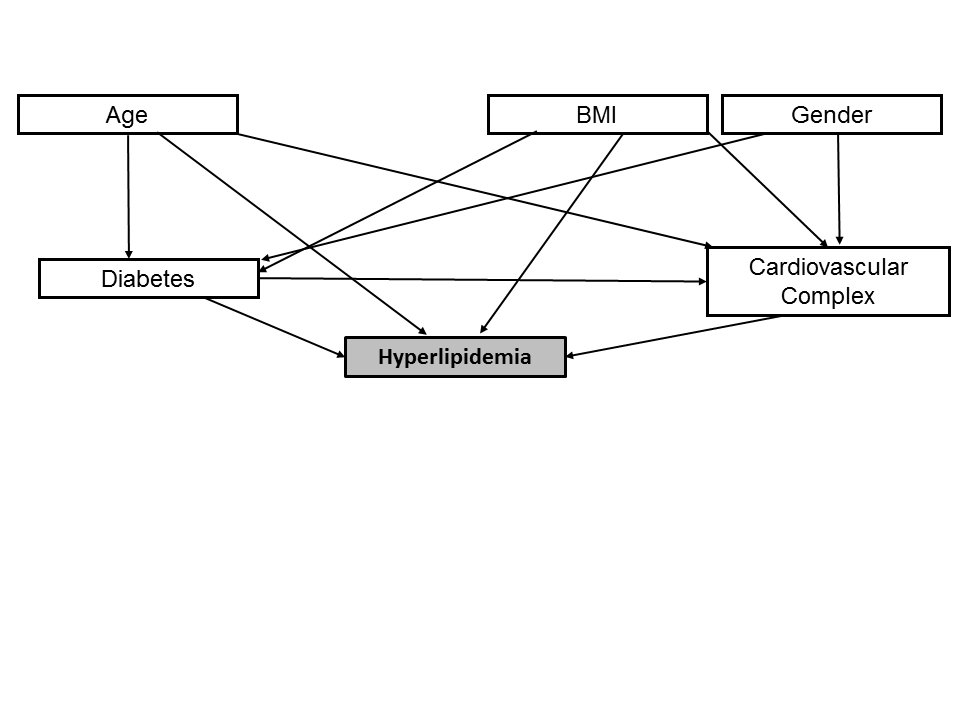

Supplement: S1 Fig — Preliminary path analysis model comprising two layers, on the top risk factors and as intermediate layer comorbidities. All of the relationships shown were statistically significant (p<0.05 each). Error terms of dependent variables have been omitted for the sake of clarity. There were no significant correlations between the independent variables. (TIF) [file pone.0177501.s001.tif]

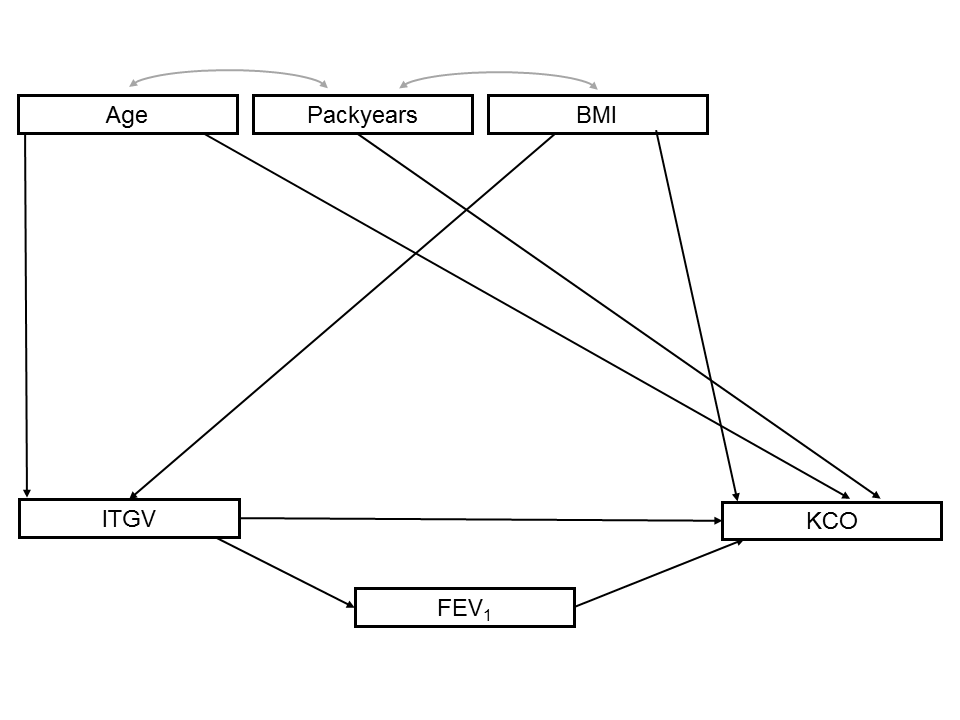

Supplement: S2 Fig — Preliminary path analysis model comprising two layers, on the top risk factors and as bottom layer lung function parameters. All of the relationships shown were statistically significant (p<0.05 each). Error terms of dependent variables have been omitted for the sake of clarity. There were no significant correlations between the independent variables. (TIF) [file pone.0177501.s002.tif]
