# Supplementary material for: Relationship of hyperlipidemia to comorbidities and lung function in COPD: Results of the COSYCONET cohort
Source: PLoS One. 2017 May 15;12(5):e0177501. doi: 10.1371/journal.pone.0177501 (PMC5432186; doi:10.1371/journal.pone.0177501)
Supplement: S1 Table — The table shows mean values and standard deviations or absolute numbers. Lung function parameters are given in terms of %predicted, except for alveolar volume, VA, which is given in liters. Column 4 shows the results of comparisons between the hyperlipidemia group (extended definition) and the complementary group of non-hyperlipidemia patients. The comparisons between groups were performed by unpaired t-tests, either for equal or unequal variances depending on the data, or by chi-square-tests in the case of categorical variables. The results of t-tests were checked by the Mann-Whitney-U-test to accommodate for deviations from normality; the results of both approaches were qualitatively equivalent. Significant (p<0.05) differences are marked with (*). (DOCX) [file pone.0177501.s003.docx]

**Table S1: Baseline characteristics of the subgroups with and without hyperlipidemia (n=2238, total cohort GOLD 1-4)**

| Parameter | All patients | Hyperlipidemia  (extended def.) | Non-hyperlipidemia | p-values |
| --- | --- | --- | --- | --- |
| N(%) | 2238 | 956 (42.7%) | 1282 (57.3%) | - |
| Gender (m/f) | 1366/872 | 619/337 | 747/535 | p<0.001* |
| Age (y) | 65.0 (±8.6) | 66.5 (±7.9) | 64.3 (±8.7) | p<0.001* |
| BMI (kg/m²) | 26.6 (±5.2) | 27.4 (±5.1) | 26.1 (±5.2) | p<0.001* |
| Waist circumf. (cm) | 99.1 (±15.6) | 101.7 (±15.2) | 97.1 (±15.5) | p<0.001* |
| Packyears | 49.1 (±35.7) | 51.8 (±36.8) | 47.1 (±35.8) | p=0.004* |
| Hb (mg/dl) | 14.68 (±1.37) | 14.57 (±1.44) | 14.76 (±1.32) | p=0.002* |
| Creatinine (mg*/*dl) | 0.89 (±0.26) | 0.92 (±0.26) | 0.87 (±0.25) | p<0.001* |
| Triglycerides (mg/dl) | 140.4 (±116.0) | 157.1 (±30.4) | 127.9 (±102.2) | p<0.001* |
| Total cholesterol (mg/dl) | 216.2 (±44.0) | 211.9 (±48.5) | 219.4 (±40.0) | p<0.001* |
| LDL (mg/dl) | 127.6 (±38.5) | 124.0 (±41.7) | 130.4 (±35.7) | p<0.001* |
| HDL (mg/dl) | 65.5 (±21.8) | 63.5 (±21.8) | 67.0 (±21.6) | p<0.001* |
| FEV_1_%pred | 55.7 (±19.4) | 57.8 (±19.3) | 54.2 (±19.3) | p=0.001* |
| FEV_1_/FVC | 54.9 (±13.7) | 54.9 (±13.4) | 54.8 (±13.8) | p=0.968 |
| FVC%pred | 78.3 (±19.1) | 77.4 (±19.3) | 79.0 (±18.9) | p=0.049* |
| TLC%pred | 111.5 (±30.0) | 110.5 (±29.2) | 112.3 (±30.6) | p=0.145 |
| RV%pred | 154.8 (±46.6) | 149.1 (±43.7) | 159.0 (±48.2) | p<0.001* |
| ITGV%pred | 150.2 (±35.6) | 145.5 (±34.6) | 153.8 (±35.9) | p<0.001* |
| VA (liter) | 5.1 (±1.2) | 5.1 (±1.2) | 5.1 (±1.2) | p=0.817 |
| TLCO%pred | 51.1 (±20.2) | 53.1 (±19.9) | 49.6 (± 20.2) | p<0.001* |
| KCO%pred | 64.8 (±23.2) | 67.7 (±22.9) | 62.5 (±23.1) | p<0.001* |
| GOLD 1/2/3/4 | 275/1001/782/180 | 132/452/315/57 | 143/549/467/123 | p=0.001* |
| GOLD A/B/C/D | 244/1175/39/768 | 100/495/16/346 | 140/680/23/425 | p=0.285 |

The table shows mean values and standard deviations or absolute numbers. Lung function parameters are given in terms of %predicted, except for alveolar volume, VA, which is given in liters. Column 4 shows the results of comparisons between the hyperlipidemia group (extended definition) and the complementary group of non-hyperlipidemia patients. The comparisons between groups were performed by unpaired t-tests, either for equal or unequal variances depending on the data, or by chi-square-tests in the case of categorical variables. The results of t-tests were checked by the Mann-Whitney-U-test to accommodate for deviations from normality; the results of both approaches were qualitatively equivalent. Significant (p<0.05) differences are marked with (*).
