# Supplementary material for: Relationship of hyperlipidemia to comorbidities and lung function in COPD: Results of the COSYCONET cohort
Source: PLoS One. 2017 May 15;12(5):e0177501. doi: 10.1371/journal.pone.0177501 (PMC5432186; doi:10.1371/journal.pone.0177501)
Supplement: S1 Text — (DOCX) [file pone.0177501.s004.docx]

**Supplement/ Supporting information**

**Procedure of path analysis**

Path analysis is primarily a method to exclude inadequate models as there are always statistical equivalent adequate models [[1](#_ENREF_24)]. Therefore a sensible model has to be constructed on the basis of previous knowledge which is derived from both conventional statistical analyses and relationships suggested by causal mechanisms. Following this reasoning we constructed path analysis models of increasing complexity, using both the results of the linear or logistic regression analyses and pathophysiological considerations. For fitting the models we used maximum likelihood estimation and checked the results by asymptotically distribution-free estimation, which was possible due to the large sample size [[1](#_ENREF_24)]. The fit of the final model was also evaluated by the bootstrap method, specifically the Bollen-Stine statistics [[1](#_ENREF_24)]. For path analysis all continuous variables were expressed as quartiles in order to reduce possible effects of peculiar distributions of single variables. Moreover, to reduce the interference with gender, quartiles were computed separately for males and females and subsequently pooled.

**Results of linear and logistic regression analyses**

To construct the path analysis model we performed conventional multivariate analyses between different sets of variables. As a first step we determined the relationship between risk factors and comorbidities. For this purpose the statistical dependence of hyperlipidemia, diabetes and cardiovascular complex on the predictors age, BMI, packyears and gender was evaluated via binary logistic regression analysis (Table A). Hyperlipidemia significantly depended on BMI and age (p<0.001 each), diabetes on BMI, gender and age (p≤0.001 each), and cardiovascular complex on BMI and age (p<0.001 each) as well as gender (p=0.046).

**Table A. Dependence of hyperlipidemia, diabetes and cardiovascular complex on the four selected risk factors BMI, packyears, age and gender**

| Parameter | BMI (kg/m²) | Packyears | Age (y) | Gender |
| --- | --- | --- | --- | --- |
| Hyperlipidemia | 1.05 (1.03; 1.07)** | 1.00 (1.00; 1.01) | 1.03 (1.02; 1.04)** | p=0.189 |
| Diabetes | 1.14 (1.11; 1.17)** | 1.00 (1.00; 1.01) | 1.03 (1.01; 1.05)** | p<0.001** |
| Cardiovascular Complex | 1.10 (1.08; 1.12)** | 1.00 (1.00; 1.00) | 1.06 (1.05; 1.08)** | p=0.046 |

The table shows the results of binary logistic regression analyses and illustrates the relationship between comorbidities and predictors. The table shows the Odds ratios as well as 95%-confidence intervals, except for gender as a binary variable. The associations marked with (**) were highly significant (p≤0.001).

In a similar fashion, the statistical dependence of lung function on risk factors was assessed via multivariate regression analysis (Table B). This revealed a significant (p<0.001 each) relationship between FEV_1_%predicted and the predictors age, BMI and gender but not packyears, whereas there were no significant associations with FVC%pred or FEV_1_/FVC. The same pattern as for FEV_1_%pred was observed for ITGV%pred (p<0.001 each). TLCO%pred showed a statistical dependence on BMI, packyears and gender (p<0.001 each) but not age, and KCO%pred was additionally dependent on packyears (p<0.001).

**Table B. Dependence of lung function parameters on the four selected risk factors BMI, age, packyears and gender**

| Parameter | BMI (kg/m²) | Age (y) | Packyears | Gender |
| --- | --- | --- | --- | --- |
| FEV_1_%pred | 0.39 (0.22; 0.56)** | 0.21 (0.10; 0.32)** | -0.01 (-0.03; 0.02) | p<0.001** |
| FEV_1_/FVC | 0.01 (-0.12; 0.14) | -0.04 (-0.12; 0.04) | -0.01 (-0.03; 0.01) | p=0.132 |
| FVC%pred | -0.08 (-0.25; 0.10) | -0.05 (-0.16; 0.06) | 0.01 (-0.02; 0.03) | p=0.126 |
| ITGV%pred | -2.09 (-2.39; -1.80)** | -0.47 (-0.66; -0.29)** | 0.01 (-0.03; 0.05) | p<0.001** |
| TLCO%pred | 1.07 (0.90; 1.24)** | 0.02 (-0.09; 0.12) | -0.05 (-0.08; -0.03) | p<0.001** |
| KCO%pred | 1.48 (1.29; 1.66)** | 0.34 (0.22; 0.45)** | -0.06 (-0.09; -0.04)** | p<0.001** |

The table shows the results of multivariate linear regression analyses in terms of the unnormalized regression coefficients as well as 95%-confidence intervals, except for gender as a binary variable. The associations marked with (**) were highly significant (p≤0.001). For abbreviations see text.

These analyses described the association between the set of risk factors and the sets of comorbidities and of lung function. To complete the standard approach, in the next step we assessed the relationship between comorbidities and lung function (Table C). This was done using linear multivariate regression analysis with lung function as dependent variables and comorbidities as predictors. FEV_1_%pred was related to hyperlipidemia and cardiovascular complex (p≤0.003 each), ITGV%pred to diabetes and hyperlipidemia (p<0.001 each) as well as cardiovascular complex (p=0.048), and KCO%pred to hyperlipidemia and diabetes (p<0.001 each) but not cardiovascular complex (p=0.178). Overall this was in accordance with the results of binary logistic regression analyses, with comorbidities as dependent and lung function as independent. Hyperlipidemia was significantly (p<0.001) associated with ITGV%pred, diabetes and cardiovascular complex with FEV_1_%pred and ITGV%pred (p<0.001 each) and also with KCO%pred (p=0.002 and p=0.031, respectively).

**Table C: Relationship between the comorbidities hyperlipidemia, diabetes and cardiovascular complex and the three selected lung function parameters FEV_1_%pred, ITGV%pred and KCO%pred**

| Parameter | Hyperlipidemia | Diabetes | Cardiovascular complex |
| --- | --- | --- | --- |
| FEV_1_%pred | 3.75 (1.87; 5.63)** | -1.16 (-3.91; 1.59) | -2.94 (-4.87; -1.00)* |
| ITGV%pred | -7.25 (-10.67; -3.88)** | -8.93 (-13.91; -3.95)** | -3.54 (-7.05;-0.04)* |
| KCO%pred | 3.28 (1.08; 5.47)* | 5.69 (2.48; 8.91)** | 1.56 (-0.71; 3.82) |

The table shows the results of multivariate linear regression analyses in terms of the unnormalized regression coefficients as well as 95%-confidence intervals. The associations marked with (**) were highly significant (p≤0.001) those with (*) significant (p<0.05). Please note that the predictors are binary variables and the dependent variables are continuous; thus the analysis is equivalent to an analysis of variance. For abbreviations see text.

**Results of preliminary path analysis models**

**Preliminary model for comorbidities**

The result for the first preliminary model containing top and intermediate layer, i.e. risk factors and comorbidities, is shown in S1 Fig. Based on the results of the logistic regression analyses (see above) this structure did not contain packyears as predictor, however additional relationships between diabetes and cardiovascular complex, diabetes and hyperlipidemia, and cardiovascular complex and hyperlipidemia were introduced to accommodate for established associations in line with the results of Fig 2. All of the relationships shown in S1 Fig were statistically significant (p<0.05 each), with a chi-squared of 4.67 and 5 degrees of freedom (p=0.46; please note that p-values >0.05 indicate acceptability of the model).

**S1 Fig. Premliminary path analysis model 1.** Preliminary path analysis model comprising two layers, on the top risk factors and as intermediate layer comorbidities. All of the relationships shown were statistically significant (p<0.05 each). Error terms of dependent variables have been omitted for the sake of clarity. There were no significant correlations between the independent variables.

**Preliminary model for lung function**

The second preliminary model comprised the top and bottom layers, i.e. risk factors and lung function, and is illustrated in S2 Fig. All relationships shown were statistically significant (p<0.001 each). Correlations between the predictors packyears, age and BMI were included to accommodate for their association, whereas gender was omitted as it turned out to be not significant, if associations between lung function parameters were allowed. These associations were modelled as links from ITGV to FEV_1_ and KCO, and as a link from KCO to FEV_1_. Basically we tried to incorporate the known inverse relation between lung volume and KCO, as well as lung volume and obstruction. The second model also fitted the data well, with a chi-squared of 4.67 at 5 degrees of freedom (p=0.46; see above).

**S2 Fig. Preliminary path analysis model 2.** Preliminary path analysis model comprising two layers, on the top risk factors and as bottom layer lung function parameters. All of the relationships shown were statistically significant (p<0.05 each). Error terms of dependent variables have been omitted for the sake of clarity. There were no significant correlations between the independent variables.

**Literature**

1. Hoyle RH (ed.): **Handbook of Structural Equation Modeling**; 2015.
