# Supplementary material for: Relationship of hyperlipidemia to comorbidities and lung function in COPD: Results of the COSYCONET cohort
Source: PLoS One. 2017 May 15;12(5):e0177501. doi: 10.1371/journal.pone.0177501 (PMC5432186; doi:10.1371/journal.pone.0177501)
Supplement: S1 File — (DOCX) [file pone.0177501.s005.docx]

**Funding/Support**

This work was supported by BMBF Competence Network Asthma and COPD (ASCONET) and performed in collaboration with the German Center for Lung Research (DZL). The project is funded by the German Federal Ministry of Education and Research (BMBF) with grant number 01 GI 0881; and is funded by unrestricted grants from AstraZeneca GmbH, Bayer Schering Pharma AG, Boehringer Ingelheim Pharma GmbH & Co. KG, Chiesi GmbH, GlaxoSmithKline, Grifols Deutschland GmbH, MSD Sharp & Dohme GmbH, Mundipharma

GmbH, Novartis Deutschland GmbH, Pfizer Pharma GmbH, Takeda Pharma Vertrieb GmbH & Co. KG for patient investigations and laboratory measurements.
